# Use of skimmed breast milk for an infant with a long‐chain fatty acid oxidation disorder: A novel therapeutic intervention

**DOI:** 10.1002/jmd2.12152

**Published:** 2020-07-30

**Authors:** Amy Kritzer, Stacey Tarrant, Karen Sussman‐Karten, Kimberly Barbas

**Affiliations:** ^1^ Division of Genetics and Genomics Boston Children's Hospital Boston Massachusetts USA; ^2^ Division of Gastroenterology, Hepatology, and Nutrition Boston Children's Hospital Boston Massachusetts USA; ^3^ Department of Nursing, Lactation Support Program Boston Children's Hospital Boston Massachusetts USA

**Keywords:** CACT, LC‐FAOD, modified‐fat breast milk, skimmed breast milk, triheptanoin

## Abstract

The focus of dietary therapy for long chain fatty acid oxidation disorders (LC‐FAODs) is to minimize fatty acid oxidation by avoiding fasting and providing sufficient calories. Dietary therapy involves restriction of long‐chain triglycerides (LCT), and provision of medium‐chain triglycerides as an alternate energy source. It is well established that the use of breast milk through the first year of a newborn's life has significant health benefits. While very few medical contraindications to breastfeeding exist, feeding an infant with a severe carnitine acylcarnitine translocase (CACT) deficiency typically requires cessation of breastfeeding as approximately 50% of the calories in human milk come from LCT. In this case report, we present the innovative and successful use of skimmed breast milk incorporated into the dietary management of an infant with severe CACT deficiency. Given the poor prognosis for individuals with severe CACT deficiency on standard dietary therapy, the use of skimmed breast milk represents an important measure to try to improve short‐term and long‐term outcomes. Given the many proven benefits of breast milk, this case illustrates that skimmed breast milk can be combined with appropriate fat sources to provide complete nutrition for children with severe CACT deficiency. After over 12 months on this regimen, this patient has experienced normal growth and development and has had no acute decompensations.

## 1 INTRODUCTION

Fatty acid oxidation disorders (FAODs) are a group of inborn errors of metabolism that result from deficiency of enzymes in the beta‐oxidation pathway, or impaired transport of long‐chain acyl‐coenzyme A (CoA)s across the mitochondrial membrane. To metabolize long‐chain fatty acids, long‐chain acyl‐CoAs are first conjugated to carnitine by the enzyme carnitine palmitoyltransferase I, then transported into the mitochondrion by carnitine acylcarnitine translocase (CACT). In the mitochondrial matrix, carnitine palmitoyltransferase II removes carnitine from long‐chain acyl‐CoAs, freeing them to be used in fatty acid oxidation. Fatty acid oxidation is an important source of energy during times of fasting or physiologic stress, and also an important source of energy for cardiac tissue, even at rest.^1^, ^2^ Impairment at any step of this process results in insufficient energy production, depletion of tricarboxylic acid cycle intermediates, and toxic build‐up of long‐chain fatty acids.^1^, ^2^, ^3^, ^4^, ^5^, ^6^ The clinical presentation of LC‐FAOD can range from nearly asymptomatic to severe, life‐threatening disease. Individuals with severe LC‐FAOD present within the first days of life with hypoketotic hypoglycemia, hyperammonemia, rhabdomyolysis, hypertrophic cardiomyopathy and heart failure, and encephalopathy. This is particularly true for individuals with CACT deficiency. Despite early identification with newborn screening, the mortality for CACT deficiency remains high on standard therapy with up to a 50% mortality risk in the first year of life.^1^, ^2^, ^3^, ^4^, ^5^, ^6^

Standard treatment for CACT deficiency is to minimize fatty acid oxidation by avoiding fasting, and to provide sufficient calories during stress or illness.^7^ Dietary therapy which restricts long‐chain triglyceride (LCT) intake has been shown to decrease the accumulation of potentially toxic acylcarnitine metabolites in LC‐FAOD, which impair cellular function, impede energy production, and alter electrical activity of the heart.^1^, ^2^, ^8^ Given the high LCT content of breast milk, breastfeeding has been contraindicated for infants with severe LC‐FAOD, such as CACT deficiency.^9^, ^10^ Breast milk offers multiple nutritional and immunological benefits, growth factors, and hormones to support growth and protect against infection.^11^ There are well‐established short‐ and long‐term medical and neurodevelopmental benefits for infants who receive breast milk, and the American Academy of Pediatrics recommends the provision of breast milk throughout the first year of life.^11^ In this case report, we describe the use of skimmed breast milk to enable the provision of breast milk for an infant with CACT deficiency.

Innovative techniques to separate fat from breast milk have been employed to treat other conditions. These have included manual separation after refrigeration, and siphoning skim milk from the bottom of a container. These methods eventually made way for the more efficient approach of using a laboratory‐grade centrifuge to remove fat from breast milk.^12^, ^13^, ^14^ Leading pediatric medical centers have skimmed breast milk by centrifuge to treat chylothorax in infants following cardiac surgery for more than 10 years.^15^, ^16^ The amount of residual fat can be assessed using a Creamatocrit (CRCT) method or a Human Milk Analyzer. An acceptable level of residual fat at our institution was determined to be 10 to 12 g/L by CRCT, with energy content of skimmed milk ranging from 43 to 50 kcal/100 mL.^17^ This translates into 20% to 25% of calories in the skimmed breast milk coming from residual long‐chain fat. With a goal of less than 10% of calories from long‐chain fat for patients with severe LC‐FAOD, the use of skimmed breast milk had never previously been considered.^8^

A recent study compared the macronutrient content of modified‐fat breast milk using three different methods of fat removal—refrigerated centrifuge, a portable cream separator, and manual separation after refrigeration.^17^ This study showed that the residual long‐chain fat is closer to 6% to 7% of total calories for both the refrigerated centrifuge and the portable cream separator methods, much lower than the previously presumed 20% to 25% of calories. This study validated a less expensive, more accessible option for separating fat that could make the continuation of skimmed breast milk available to settings without a centrifuge. Additionally, this study showed that protein content of the breast milk is not significantly affected by any of the fat removal processes. In a separate study, IgA levels were found to decrease slightly after refrigeration and manual fat separation or separation by centrifuge, but IgA was still present in high enough levels to provide immune benefits to the child.^13^

## 2 CASE REPORT

Baby girl JM is a 3610 g term infant born via spontaneous vaginal delivery to a 32‐year‐old mother with a history of 23‐week loss of twins. She was discharged home with parents at 24 hours of life. At home that day, she was noted to be very sleepy with low tone and no interest in breastfeeding. She became progressively more irritable and then went limp. She was transported to the hospital by emergency services where initial evaluation showed blood glucose in the 30s and core temperature of 91 °F. She was admitted to a local NICU for further evaluation and management. In the NICU, her labs showed an initial ammonia level of 170 μmol/L. She began having seizures and a repeat ammonia level was 546 μmol/L. Ammonul loading dose and continuous infusion were given and she was transferred to our level 3 NICU on day of life 3 with encephalopathy, hyperammonemia, hypertrophic cardiomyopathy (LV septal thickness z‐score + 5) and creatine kinase of 1114 units/L (normal <150). Initial state screen showed elevated C16 of 12.88 μmol/L and C18:1 of 2.85 μmol/L consistent with either CPT II deficiency or CACT deficiency. Once newborn screen was reported, Ammonul was stopped and high dextrose 20% infusion at 100 cc/kg was continued. Clinical acylcarnitine profile at the time of admission showed elevated C16 level of 9.54 μmol/L (normal <0.35) and elevated C18:1 of 1.86 μmol/L (normal <0.24). Creatine kinase on admission was 993 units/L. The preliminary diagnosis and standard dietary therapy were discussed with the parents. Given the poor outcomes with standard dietary therapy, the medical team also discussed the potential to use an investigational odd‐chain medium‐chain triglyceride (MCT), triheptanoin. Clinical trials had demonstrated that triheptanoin resulted in decreased hypoglycemia, rhabdomyolysis, and cardiomyopathy event rates as well as decreased hospitalizations and total hospital days in patients with LC‐FAOD.^18^, ^19^, ^20^ Additionally, triheptanoin had been used successfully to rescue children who developed cardiomyopathy on standard MCT therapies.^21^ Genetic testing revealed a likely pathogenic variant (c.397 C > T, p.Arg133Trp) and a variant of uncertain significance (c.658G > A, p.Gly220Arg) in SLC25A20. Parental testing confirmed these variants were in trans.

Emergency FDA approval was obtained for the compassionate use of triheptanoin. Triheptanoin cannot be combined with any other MCT product, which meant that the MCT‐based formulas previously used in babies with LC‐FAOD could not be used and a modular formula would be needed. Multidisciplinary discussions raised the question of using skimmed breast milk as the base to which triheptanoin could be added.^17^ Skimmed breast milk had been successfully used for other children who required a low fat diet, such as those with chylothorax.^15^, ^16^

JM's mother was highly motivated to express milk since it was a tangible, empowering action she could take to contribute to her daughter's treatment. As skimming breast milk results in a loss of approximately 20% to 25% of the volume when the cream is removed, in order to provide JM with exclusive human milk feedings, her mother would have to produce a surplus of milk. At day 9, she was producing between 360 and 420 mL daily. With support from the lactation team, JM's mother used techniques to optimize her use of the pump and increase pumping frequency, and at discharge, she was producing 1200 mL daily. The macronutrient composition of the skimmed breast milk obtained by a cream separator in this case was similar to samples from the refrigerated centrifuge used in the hospital as measured by CRCT and was comparable to prior published data.^17^

Given the ability to skim fat from expressed breast milk to such a low percentage, we felt confident that we could use modified‐fat breast milk, skimmed using the portable cream separator method, in the case of JM. We designed a recipe using the modified‐fat breast milk that met all the criteria for feeding an infant with a severe LC‐FAOD (Tables 1 and 2). With the modified‐fat breast milk as the base, we added soybean oil to provide essential fatty acids at 3% of calories for linoleic acid and 0.45% of calories for alpha‐linolenic acid, triheptanoin at 4 g/kg/day to provide approximately 73% of total fat intake, and protein and carbohydrate modulars to provide additional calories. She was switched to walnut oil at 2 1/2 months of age as a fatty acid profile indicated that more essential fatty acids were needed, and walnut oil is higher in essential fatty acids than soybean oil. This combination was well tolerated by the infant, with no adverse gastrointestinal symptoms.

**TABLE 1:** Macronutrient breakdown of 100 mL modified fat breast milk

Fat (g)	Protein (g)	Carbohydrate (g)
0.24	1.22	7.18

**TABLE 2:** Macronutrient breakdown of discharge formula recipe by component

	LCT (g)	MCT (g)	Protein (g)	Carbohydrate (g)
24 oz. modified fat breast milk	1.8	—	9.0	53.0
5 mL soybean oil	4.5	—	—	—
1 tbsp. + ½ tsp. protein modular	—	—	2.5	—
40 g carbohydrate modular	—	—	—	38.4
17.6 mL triheptanoin	—	16.9	—	—
Total	6.3	16.9	11.5	91.4

Abbreviations: LCT, long‐chain triglycerides; MCT, medium chain triglyceride.

**FIGURE 1:**
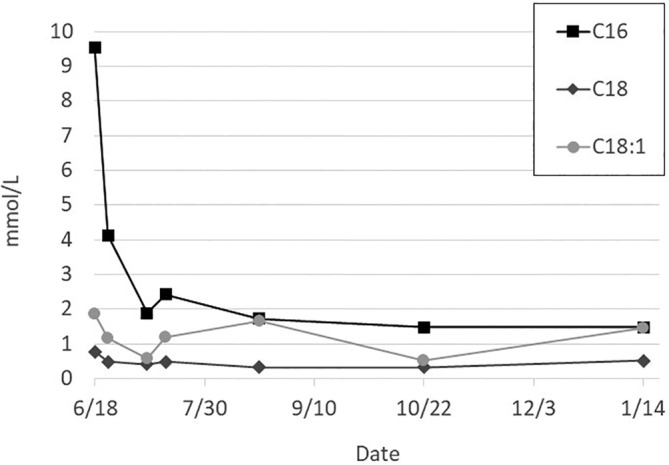
Acylcarnitine levels throughout treatment

The family purchased a portable cream separator as used in the above‐mentioned study, which is readily available for online purchase for less than $300, and they were extensively educated on its use by our lactation consultants (see Section 3). The infant was discharged from the hospital at 33 days of age, weighing 4.55 kg, and receiving 86 mL every 3 hours of the formulated skimmed breast milk. At the time of discharge, she showed significant improvement in biochemical parameters with C16 level of 2.54 μmol/L, a C18:1 level of 1.18 μmol/L, and a creatine kinase level of 92 units/L. Figure 1 shows that acylcarnitines have remained consistent throughout treatment. Creatine kinase levels have remained essentially normal as well.

**FIGURE 2:**
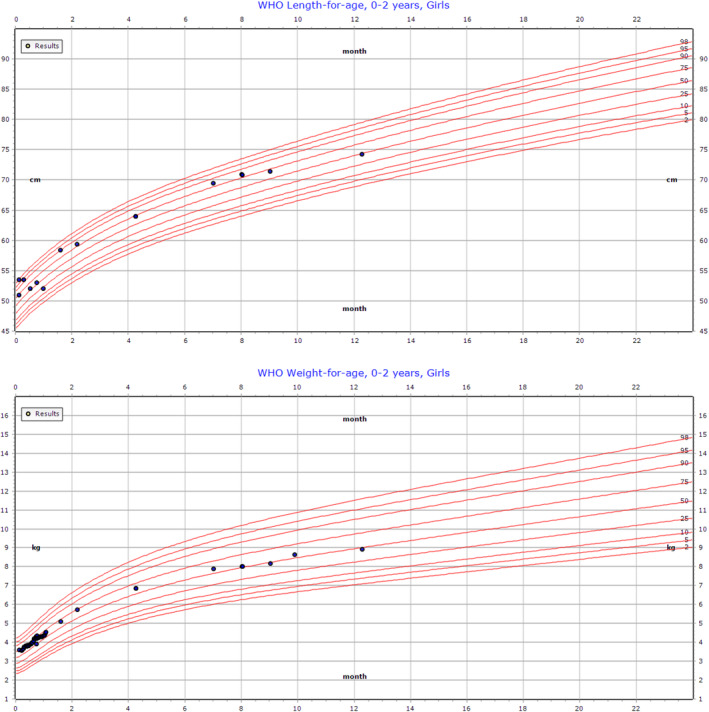
Growth parameters

She continues to demonstrate growth along the 60th to 70th percentiles (Figure 2). She has had no acute decompensations and no episodes of hypoglycemia, hyperammonemia, or rhabdomyolysis.

Nutritional parameters, including vitamin D and fatty acid profiles, were monitored and remained within normal limits. Her cardiomyopathy has resolved and she maintains normal biventricular septal thickness and function. Despite her early severe medical complications, she also has demonstrated normal gross motor, fine motor, speech, and social milestones.

## 3 METHODS

**FIGURE 3:**
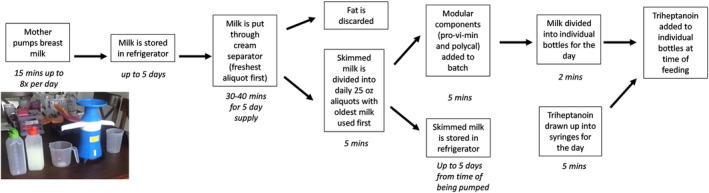
Schematic for milk separation using the portable cream separator and home setup

The macronutrient composition of the skimmed breast milk obtained by a cream separator in this case was similar to samples from the refrigerated centrifuge used in the hospital and comparable to prior published data.^17^ This family purchased a cream separator with 120 V motor and capacity to process up to 100 L/h (Figure 3). The pooled breast milk is poured into the collecting bowl and flows to separation channels between 12 layers of disks through vertically aligned distribution holes in the stack. The centrifugal force causes the milk globules to move into separating channels based on their density. The skimmed milk, with heavier density, moves outward to the skim milk separating spout. The cream from the breast milk has a lower density and moves inward on the device, to the cream spout.

## 4 DISCUSSION

To our knowledge, the use of skimmed breast milk has never been tried before in CACT deficiency or other severe LC‐FAOD, and thus represents an important advancement in the care of these infants. Breast milk is high in long‐chain fats and is unsuitable for infants with LC‐FAOD. However, skimming breast milk to remove long‐chain fats allows supplementation with a custom regimen of medium‐chain fats while also providing the additional health benefits of breast milk. As acute illness is a major trigger for decompensation in infants with CACT deficiency,^21^ the added immunological benefits are especially important. In addition, specialized formulas that are typically used for infants with LC‐FAOD cannot be supplemented with specialized MCT, such as the triheptanoin as in JM's case. Thus, the use of skimmed breast milk provides greater flexibility for nutritional therapy and would allow for use of the newly FDA‐approved triheptanoin.

One limitation to the use of skimmed breast milk is that it adds complexity to the feeding regimen when compared to the use of an intact formula with low long‐chain fat and high MCT. A portable cream separator must be purchased, as the manual method of separating fat from breast milk after refrigeration has shown insufficient and inconsistent removal of fat.^17^ While more affordable than a laboratory centrifuge, the cost of the portable cream separator may not be affordable for some families and would not likely be covered by insurance. Batches of breast milk must then be skimmed, and the family must have adequate storage for skimmed milk that will not be immediately used. The use of skimmed breast milk also requires that additional components must be accurately measured and added to the skimmed breast milk to provide complete nutrition. This regimen might prove difficult for families with limited social, educational, or financial resources. In some situations, pumping breast milk can be labor intensive and the ability to sustain adequate production for skimming over the long term may not be feasible.

While there are limitations, there are clear health advantages to allowing a baby to receive its mother's milk. Additionally, it allows us to meet the mother's personal goals and the public health goals for breastfeeding. Including parents in discussions around options for innovative care is an essential part of family‐centered care. The opportunity to provide skimmed breast milk has been incredibly empowering for this family. It has also allowed us to provide triheptanoin, which has been promising in improving health outcomes in individuals with LC‐FAOD in multiple clinical trials.^18^, ^19^, ^22^

## 5 CONCLUSION

This case report demonstrates that skimmed breast milk can be successfully incorporated into the dietary management of an infant with severe CACT deficiency, with adequate growth and beneficial health outcomes. It also demonstrates that breast milk can be successfully used with triheptanoin. We speculate that this regimen would also be beneficial for infants with other severe disorders of LC‐FAOD.

## CONFLICT OF INTEREST

A. K. served as a consultant for Ultragenyx in June 2020. S. T., K. B., and K. S.‐K. declare that they have no conflicts of interest.

## AUTHOR CONTRIBUTIONS

All authors contributed to the design, implementation, and execution of this study. All authors contributed to the writing and editing of the manuscript.

## INFORMED CONSENT

All procedures followed were in accordance with the ethical standards of the responsible committee on human experimentation (institutional and national) and with the Helsinki Declaration of 1975, as revised in 2000 (5). Informed consent was obtained from all patients for being included in the study. Additional informed consent was obtained from all patients for which identifying information is included in this article.
